# Effects of SARS-CoV-2 infection on human reproduction

**DOI:** 10.1093/jmcb/mjab025

**Published:** 2021-05-18

**Authors:** Ming Yang, Jing Wang, Yidong Chen, Siming Kong, Jie Qiao

**Affiliations:** 1 Center for Reproductive Medicine, Department of Obstetrics and Gynecology, Peking University Third Hospital, Beijing Key Laboratory of Reproductive Endocrinology and Assisted Reproductive Technology, Beijing 100191, China; 2 National Clinical Research Center for Obstetrics and Gynecology, Beijing 100191, China; 3 Key Laboratory of Assisted Reproduction (Peking University), Ministry of Education, Beijing 100191, China; 4 Beijing Advanced Innovation Center for Genomics, Beijing 100871, China; 5 Peking-Tsinghua Center for Life Sciences, Peking University, Beijing 100871, China; 6 Research Units of Comprehensive Diagnosis and Treatment of Oocyte Maturation Arrest, Peking University, Beijing 100871, China; 7 Academy for Advanced Interdisciplinary Studies, Peking University, Beijing 100871, China

**Keywords:** COVID-19, SARS-CoV-2, reproduction, vertical transmission

## Abstract

The worldwide infection of severe acute respiratory syndrome coronavirus 2 (SARS-CoV-2) impacts human health and life on multiple levels. People infected with SARS-CoV-2 suffer from physical disorders and psychological distress. At present, no direct evidence indicates that SARS-CoV-2 negatively influences human reproduction, and the possibility that gametes and embryos are affected requires further investigation. To evaluate the potential effects of SARS-CoV-2 infection on human reproduction and fetal health, this review summarizes the basic and clinical research of SARS-CoV-2 on reproduction up to date, hoping to offer guidance and advice to people at reproductive age and provide clues for the prevention and treatment of associated diseases.

## Introduction

Since the first identification of coronavirus disease-19 (COVID-19) in December 2019 (Chen et al., 2020a), the global spread of severe acute respiratory syndrome coronavirus 2 (SARS-CoV-2) has posed a major threat to the public health system. The confirmed cases reported around the world have exceeded 110 million until March 12, 2021, and the number of deaths is over 2 million. This is the third coronavirus infection outbreak of the 21st century. Every country has adopted various strategies to combat the pandemic and to restore the world’s prosperity. The incubation period of SARS-CoV-2 infection is 1–14 days (Lin and Li, 2020). Fever, dry cough, nasal congestion, runny nose, sore throat, myalgia, and pneumonia are the main clinical symptoms. Diarrhea or conjunctivitis is the first symptom in a small number of patients (Mehta et al., 2020; Chen et al., 2020a). Among them, the elderly with chronic diseases is more likely to become critically ill. No effective medicine specific for COVID-19 has been developed yet.

Zinc metallopeptidase angiotensin-converting enzyme 2 (ACE2), first discovered in 2000 (Zisman et al. 2003), is a cellular receptor for SARS-CoV and SARS-CoV-2 (Turner et al., 2004; Wan et al., 2020). *ACE2* is expressed in many systems (Zou et al., 2020) and correlates with several tissue functions (Tikellis et al., 2003; Zisman et al., 2003). Lung alveolar epithelial cells, enterocytes of the small intestine (Hamming et al., 2004), type II alveolar cells (AT2) (Zhao et al., 2020), respiratory epithelial cells, myocardial cells, epithelial cells of ileum and esophagus, proximal tubule cells of kidney, and bladder urothelial cells (Zou et al., 2020) all express *ACE2*. Organs and tissues with ACE2 are believed to have a higher susceptibility to SARS-CoV-2 infection (Zou et al., 2020). The analysis of *ACE2* expression patterns in embryos and gonads is valuable for further investigate into potential effects and mechanisms of SARS-CoV-2 on reproduction.

**Figure 1:**
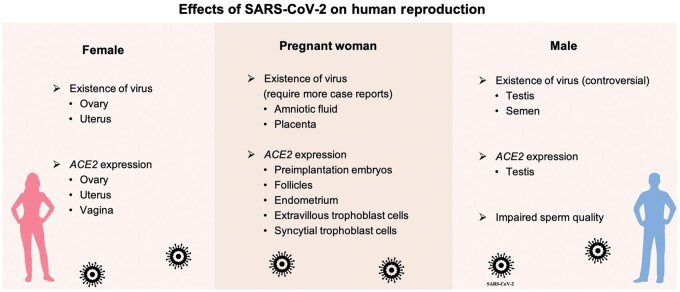
Effects of SARS-CoV-2 on human reproduction.

Autopsy results have showed the presence of SARS-CoV-2 in the reproductive system of infected patients (Bian and The COVID-19 Pathology Team, 2020; Ma et al., 2021). Whether SARS-CoV-2 affects human reproduction and embryonic development remains to be clarified. Therefore, this review hopes to give some insights into the effects of SARS-CoV-2 infection on human reproduction (Figure 1).

## Impact on the female reproductive system

The female reproductive system is composed of ovaries, fallopian tubes, and uterus. The functional cooperation of these organs allows steroid hormones production, oocytes maturation, successful fertilization, as well as embryo development (Mancini and Pensabene, 2019). The molecular functions of ACE2, angiotensin II (AngII), Ang-(1–7), and mitochondrial assembly (Mas) are connected closely (Santos et al., 2003, 2013; Turner et al., 2004). *ACE2* and *Mas* are expressed in human ovaries (Reis et al., 2011). Previous studies reported that Ang-(1–7) and Mas co-localized to primordial, primary, secondary, and antral follicles and were present in the stroma and corpora lutea of human reproductive-aged ovaries; Ang-(1–7) was also detectable in follicular fluid.

To our knowledge, the female reproductive system is susceptible to many other viruses, such as HSV-2, HIV, and Zika virus (Counotte et al., 2018; Keller et al., 2019). Bian and The COVID-19 Pathology Team (2020) detected SARS-CoV-2 in uterus and ovary by polymerase chain reaction (PCR), immunohistochemistry, and transmission electron microscopy (TEM) for the first time. Vaginal swab samples from 10 women diagnosed with COVID-19 were collected and proved to be negative for SARS-CoV-2 (Qiu et al., 2020). In 35 female severe COVID-19 patients who were in the postpartum and postmenopausal stage, no presence of the virus in vaginal fluid and exfoliated cells was found (Cui et al., 2020). Considering the limited sample size, an infection risk of female reproductive system and the possibility of vertical transmission of SARS-CoV-2 cannot be completely ruled out.

SARS-CoV-2 needs entry factors ACE2 (Turner et al., 2004; Wan et al., 2020), TMPRSS2 (Djomkam et al., 2020), CatB/L (Djomkam et al., 2020), and CD147 (Wang et al., 2020a) to promote its invasion. In theory, organs with a high expression level of cell entry factors can be susceptible to SARS-CoV-2. *ACE2* has been confirmed to express widely in ovaries, uterus, and vagina in the female reproductive system. In the uterus, Vaz-Silva et al. (2009) found a higher *ACE2* expression in epithelial cells compared to stromal cells and in secretory phase compared to proliferative phase. Moreover, data from the Human Protein Atlas and GeneCards also confirmed the presence of ACE2 in the uterus and vagina (Yan et al., 2020). On the contrary, at a single-cell level, the results appeared to be different. Single-cell sequencing data from normal cells in ovaries, fallopian tube, and uterus showed no significant expression of either *ACE2* or *TMPRSS2*. The co-expression of *ACE2* with *TMPRSS2*, *CTSB*, and *CTSL* was also not detected (Goad et al., 2020). Therefore, it might be hard for SARS-CoV-2 to attack normal uterus and vagina cells. These organs tend to be strong against infection, which could also explain the absence of the virus reported in some studies. Henarejos-Castillo et al. (2020) studied endometrium gene expression throughout the menstrual cycle from 112 COVID-19 patients. The findings showed that although low expression levels of *ACE2* and *TMPRSS2* suggested a safe environment against the virus entry into host cells, expression levels of proteases such as *TMPRSS4*, *CTSB*, and *CTSL* significantly increased during the early and middle secretory phases, which might confer a susceptibility of infection through different mechanisms. In addition, virus-related gene expression was shown to increase with age, suggesting higher risk of infection to the reproductive system for older women (Henarejos-Castillo et al., 2020).

Overall, the existing studies are limited by small sample sizes and individual differences. The potential influences of the virus require further validation. More completed and detailed diagnoses and autopsies of female reproductive organs in COVID-19 patients may provide more insights to explore the true influence of the virus.

## Impact on the male reproductive system

Currently, the infection of SARS-CoV-2 in the male reproductive system is still uncertain and controversial. Majority of the researches revealed that no SARS-CoV-2 was detected in testes or semen in COVID-19 patients (Guo et al., 2020; Ruan et al., 2021). Nevertheless, three researches reported the detection of SARS-CoV-2 in testes and semen of COVID-19 patients (Yang et al., 2020; Li et al., 2020a; Ma et al., 2021). To be specific, 6 of 38 COVID-19 patients were detected SARS-CoV-2-positive in semen (Li et al., 2020b); 1 of 12 COVID-19 patients was detected SARS-CoV-2-positive in testes of postmortem examination (Yang et al., 2020); and SARS-CoV-2 spike S1 protein was stained positive in COVID-19 patients’ testes (Ma et al., 2021). Considering the risk of sample contamination, some researchers were skeptical to SARS-CoV-2-positive test in semen (Paoli et al., 2020a, b). Up to now, no study has reported the presence of SARS-CoV-2 in the prostate. TMPRSS2 exists in different parts and cells of normal prostate and prostate cancer (Afar et al., 2001), so the possibility of prostatitis caused by SARS-CoV-2 cannot be excluded. More details about the SARS-CoV-2 existence in the male reproductive system are shown in Table 1.

**Table 1:** Studies of impacts on male reproductive system.

Study	Sample type	Number of sample	Infection stage of sample collection	SARS-CoV-2 test	Sperm quality	Others
Yang et al. (2020)	Testis	12	Autopsy	1 positive	NR	COVID-19 patients testes: seminiferous tubular injury; Leydig cells reduced
Li et al. (2020a)	Testicular/epididymal specimens	6	Autopsy	NR	NR	Seminiferous tubules thinning; higher apoptotic cell numbers within seminiferous tubules
Pan et al. (2020)	Semen	34	Median: 31 days from COVID-19 diagnosis	All negative	NR	19% of patients in our cohort had scrotal discomfort
Kayaaslan et al. (2020)	Semen	16	All in accute stage	All negative	NR	NR
Song et al. (2020)	Testis/semen	1 testis/12 semen	Autopsy (testis); 1 in infection stage; 11 in recovery stage	All negative	NR	NR
Guo et al. (2020)	Semen	23	12 in infection stage; 11 in recovery stage	All negative	Total sperm counts, total motile sperm counts, and sperm morphology	NR
Paoli et al. (2020a)	Semen	1	All in infection stage	Negative	NR	NR
Ma et al. (2020b)	Semen	12	1 in infection stage; 11 in recovery stage	All negative	8 with normal sperm parameters and low DFI; 4 with low sperm motility with higher sperm DFI (2 with poor sperm morphology)	NR
Zhang et al. (2020)	Prostatic secretion	10	3 in infection stage; 7 in recovery stage	All negative	NR	NR
Ruan et al. (2021)	Urine/semen	74 urine/70 semen	All in recovery stage	Urine 0/74 positive; semen 0/70 positive	Lower total sperm count with a long time (≥90 days) since recovery	NR
Ning et al. (2020)	Semen	17	9 in infection stage; 8 in recovery stage	All negative	NR	Orchidoptosis of 3 severe COVID-19 patients (2.7%)
Holtmann et al. (2020)	Semen	18	All in recovery stage	All negative	Sperm concentration/count/progressive motility decreased in morderate group	NR
Li et al. (2020b)	Semen	38	15 in accute stage; 23 in recovery stage	4/15 positive in accute stage; 2/23 positive in recovery stage	NR	NR

NR, not reported.

Notably, orchitis was found in the testicles of male COVID-19 patients through autopsy (Duarte-Neto et al., 2020), raising the question of whether SARS-CoV-2 affects sperm quality. A research reported that all five cases of male patients with COVID-19 had suffered from severe spermatogenesis damage compared with the normal control group. In addition, the study also observed significant infiltration of immune cells in testes of COVID-19 patients (Ma et al., 2021). Holtmann et al. (2020) reported that, compared with the mildly infected group and the control group, sperm quality (sperm concentration, counts of sperm per ejaculate, counts of progressive motility, and counts of complete motility) was significantly different in the moderately infected group. Moreover, although the differences in volume, complete motility, and amount of immotile sperms were of statistical significance between fever-positive and fever-negative groups, the values were still within normal range. Segars et al. (2020) indicated declined sperm concentration and motility for 72–90 days due to fever after SARS-CoV-2 infection.

Pathological examinations and hormones were also studied in COVID-19 patients. Pathological examinations in infected males showed a series of changes in seminiferous tubules. Sertoli cells displayed severe injuries, including swelling, vacuolation, cytoplasmic rarefaction, and detachment from tubular basement membranes. Cell clusters in the lumen also decreased and shed in number. Leydig cells reduced and the basement membrane thickened with peritubular fibrosis along with mild inflammatory infiltration in the interstitium (Deshmukh et al., 2020; Yang et al., 2020). SARS-CoV-2 infection may also affect male hormone production. Schroeder et al. (2020) found lower testosterone and dihydrotestosterone levels in most male participants with intensive care. Rastrelli et al. (2020) reckoned that most COVID-19 patients had lower total testosterone levels. While serum luteinizing hormone (LH) was higher in the 81 infected patients, testosterone to LH (T/LH) ratio and follicle-stimulating hormone (FSH) to LH (FSH/LH) ratio were lower. The T/LH ratio in COVID-19 patients was negatively associated with disease severity, aspartate transaminase concentration, and C-reactive protein levels and was positively associated with serum anti-müllerian hormone level (Ma et al., 2020).

There is still no strong evidence to support that COVID-19 infection directly leads to male infertility. To understand long-term influences of SARS-CoV-2 on the male reproductive system, more thorough studies are needed.

## Vertical transmission of pregnant women with COVID

Autopsy of COVID-19 victims in China has confirmed SARS-CoV-2 infection in uterus and ovaries (Bian and The COVID-19 Pathology Team, 2020), indicating the possible severe scenario of vertical transmission. It is worth noting that using TEM, Algarroba et al. (2020) first found the virus invading human placenta, but the infant was tested negative for SARS-CoV-2. SARS-CoV-2 was also identified to localize predominantly in syncytiotrophoblast cells (Hosier et al., 2020), amniotic and placental (Richtmann et al., 2020). In addition, compared to healthy individuals, the placentas of infected pregnant women have common abnormalities such as villous agglutination, subchorionic thrombi, focal avascular villi, and thrombi in larger fetal vessels (Mulvey et al., 2020; Smithgall et al., 2020; Dong et al., 2020a; Zeng et al., 2020a). In a study that 29 infected women accepted SARS-CoV-2 detection by reverse transcription (RT)–PCR using their amniotic fluid, placenta, breast milk, and cord blood, all samples were negative (Schwartz, 2020). Throat swab samples of 16 newborns delivered by 15 infected pregnant women were tested negative for SARS-CoV-2 (Zhu et al., 2020; Chen et al., 2020a). However, in one case, nasopharyngeal swabs obtained from the neonate on the day of birth, Day 2, and Day 7 were all SARS-CoV-2-positive, neonatal plasma tested positive on Day 4, and stool was positive on Day 7 (Kirtsman et al., 2020). In addition, some neonates were confirmed with SARS-CoV-2 infection at 16 h (Alzamora et al., 2020), 24 h (Sisman et al., 2020; Zeng et al., 2020b), and 36 h (Marzollo et al., 2020; Yu et al., 2020; Wang et al., 2020b) after birth. Although placenta, cord blood, and breast milk were tested negative (Yu et al., 2020; Wang et al., 2020b) and the detection was not carried at once after birth, the possibility of vertical transmission cannot be excluded. Moreover, two studies reported that IgM and IgG antibodies of novel coronavirus existed in the newborns, but the virus was tested negative in the fetus (Zeng et al., 2020a; Dong et al., 2020b). Since IgM antibodies generally cannot be transmitted through the placenta to the fetus and the production of IgM usually takes 3–7 days after infection, there might be an intrauterine infection. Another study reported 3 of 11 neonatal placentas tested positive for SARS-CoV-2, but the neonatal virus test was negative (Penfield et al., 2020).

A recent study reported for the first time that SARS-CoV-2 was detected to transmit from mother to child through the placenta (Vivanti et al., 2020). A 23-year-old pregnant woman with a fever (38.6°C) and severe cough at 35 + 2 weeks of pregnancy tested positive for SARS-CoV-2 in blood, throat swabs, and vaginal swabs. Five days later, a cesarean section was performed. During the cesarean section, transparent amniotic fluid was collected before rupture of the amniotic membrane, and it was positive for SARS-CoV-2. The mother was discharged from the hospital 6 days after delivery and was in good condition. Six hours after birth and extubation, the newborn’s blood, non-bronchial, and bronchoalveolar lavage fluid were found SARS-CoV-2-positive. Throat swab and rectal swab were collected after cleaning the newborn 1 h after birth, and then repeated on Day 3 and Day 18 after birth: SARS-CoV-2 tests were all positive. RT–PCR of two SARS-CoV-2 genes in the placenta showed positive results. Therefore, vertical transmission of SARS-CoV-2 infection was suggested to exist in this case (Vivanti et al., 2020).

However, this case only confirmed SARS-CoV-2 vertical transmission in the third trimester. Due to a paucity of evidence, caution should be undertaken to draw the link between vertical transmission and SARS-CoV-2 infection. Pregnant women with COVID-19 may have risk of death, premature delivery, and infection of newborns. As such, pregnant women with COVID-19 need to be closely observed and cared for. More details about the outcome of COVID-19 pregnant women and newborns are shown in Table 2.

**Table 2:** Case outcomes of pregnant women with COVID-19.

Study	Number of mothers with COVID	Number of newborn	Gestational age (weeks + days)	Vaginal delivery (VD) or cesarean section (CS)	Preterm deli very	Death of mother	SARS-CoV-2 test						Newborn throat swab	Newborn stool	Newborn blood	Neonatal sero logical test	Anal swab	Death of new born
							Amniotic fluid	Placenta	Cord blood	Breast milk	Maternal blood	Vaginal						
Chen et al. (2020a)	9	9	Median 37 + 2	CS 9	4/9	0	Negative	NR	Negative	Negative	NR	NR	Negative	NR	NR	NR	NR	0
Wang et al. (2020b)	1	1	40	CS	0	0	NR	Negative	Negative	Negative	NR	NR	Positive at 36 h after birth	NR	NR	NR	NR	0
Wang et al. (2020c)	1	1	30	CS	1	0	Negative	Negative	Negative	NR	NR	NR	Negative	NR	NR	NR	NR	0
Li et al. (2020d)	16	17	Median 38	CS 14	3/16	0	NR	NR	NR	NR	NR	NR	Negative	NR	NR	NR	NR	0
Kirtsman et al. (2020)	1	1	35 + 5	CS	1	0	NR	NR	Negative	NR	NR	NR	Positive on Day 1/2/7 after birth	Positive on Day 7 after birth	Positive on Day 4	NR	NR	0
Hantoushzadeh et al. (2020)	9	7	Second or third trimester	CS 6; VD 1	6/7	7/9	NR	NR	NR	NR	NR	NR	Negative 5/5	NR	NR	NR	NR	2/7
Fan et al. (2021)	2	2	36 + 5 and 39	CS 2	1/2	0	Negative	Negative	Negative	Negative	Negative	Negative	Negative	NR	NR	NR	NR	0
Li et al. (2020e)	1	1	35	CS	1	0	NR	NR	NR	NR	NR	NR	Negative	Negative	Negative	NR	NR	0
Dong et al. (2020b)	1	1	38	CS	0	0	NR	NR	NR	NR	NR	NR	Negative	NR	NR	NR	NR	0
Peng et al. (2020a)	1	1	35	CS	1	0	Negative	Negative	Negative	Negative	NR	Negative	Negative at 2 h and on Day 1/2/3/7/14 after birth	Negative on Day 1/7 after birth	NR	NR	NR	0
Marzollo et al. (2020)	1	1	38	VD	0	0	NR	NR	NR	NR	NR	NR	Positive at 36 h after birth	NR	NR	NR	NR	0
Yu et al. (2020)	7	7	Median 39 + 1	CS 7	0	0	NR	NR	NR	NR	NR	NR	1 positive at 36 h after birth	NR	NR	NR	NR	0
Sisman et al. (2020)	1	1	34	VD	1	0	NR	NR	NR	NR	NR	NR	Positive at 24/48 h after birth	NR	NR	NR	NR	0
Demirjian et al. (2020)	1	1	39	CS	0	0	NR	NR	NR	NR	NR	NR	Negative within 24 h; positive on Day 3 after birth	Negative within 24 h and Day 3 after birth	Negative within 24 h and Day 3 after birth	NR	NR	0
Zhu et al. (2020)	9	10	Median 34 + 5	CS 7	6/9	0	NR	NR	NR	NR	NR	NR	Negative	NR	NR	NR	NR	1/10
Polónia-Valente et al. (2020)	1	1	38	VD	0	0	NR	NR	NR	NR	NR	NR	Negative at 2/48 h after birth	NR	NR	NR	NR	0
Cooke et al. (2020)	2	2	28–29	CS 2	2	0	NR	NR	NR	NR	NR	NR	Negative 2/2	NR	NR	NR	NR	0
Peng et al. (2020b)	1	1	38	CS	0	0	NR	NR	NR	NR	NR	NR	Negative on Day 3 after birth	NR	NR	Positive IgG; negative IgM	NR	0
Zheng et al. (2020)	2	2	36–39	CS 2	1/2	0	NR	NR	NR	NR	NR	NR	Negative 2/2	NR	NR	NR	Negative 2/2	0
Alzamora et al. (2020)	1	1	33	CS	1	0	NR	NR	NR	NR	NR	NR	Positive at 16/48 h after birth	NR	NR	Negative IgG/IgM	NR	0
Reis et al. (2020)	3	3	28–40	CS 3	2/3	1/3	NR	NR	NR	NR	NR	NR	Negative 3/3	NR	NR	NR	NR	0
Grimminck et al. (2020)	1	1	38	VD	0	0	NR	Negative	NR	NR	NR	Negative	Negative	NR	NR	NR	NR	0
Lv et al. (2020)	1	1	31	CS	1	0	Negative	Negative	Negative	NR	NR	NR	NR	NR	NR	NR	Negative	0
Zeng et al. (2020b)	NR	33	NR	NR	NR	NR	NR	NR	NR	NR	NR	NR	Positive 3/33	NR	NR	NR	NR	0
Liu et al. (2021)	13	13	Median 35	CS 10	6/13	0	NR	NR	NR	NR	NR	NR	NR	NR	NR	NR	NR	1/13
Chen et al. (2020c)	4	4	Median 36 + 6	CS 3	0	0	NR	NR	NR	NR	NR	NR	Negative	NR	NR	NR	NR	0
Hecht et al. (2020)	20	21	30–41	CS 12	9/20	0	NR	Positive	NR	NR	NR	NR	Positive 1/9 on Day 2/4 after birth	NR	NR	NR	NR	1/10

NR, not reported.

## The potential mechanism of SARS-CoV-2 affecting the reproductive system

ACE2 and TMPRSS2 are important for SARS-CoV-2 invasion (Djomkam et al., 2020). *ACE2* is expressed in several spermatogenic cell types, with Sertoli cells having the highest expression level. *TMPRSS2* is also expressed in the male reproductive system, but has a different expression pattern compared to *ACE2* (Liu et al., 2020a), suggesting the potential danger of SARS-CoV-2 to spermatogenesis.

Because the blood–testis barrier cannot completely block the virus, males infected by SARS-CoV-2 would present an increase in LH and a decrease in T/LH ratio and FSH/LH ratio (Ma et al., 2020; Wang et al., 2020a). Furthermore, COVID-19 induced oxidative stress at cellular level, leading to sperm DNA fragmentation and reduced sperm motility (Homa et al., 2019; Anifandis et al., 2020). SARS-CoV-2 infection can cause fever and elevated testicular temperature (Li et al., 2020c), which may impair spermatogenesis (Jung and Schuppe, 2007). Male patients with severe COVID-19 who have a secondary cytokine storm syndrome (hemophagocytic lymphohistiocytosis) (Mehta et al., 2020) may undergo immunomodulatory therapy; the patient might experience a deviation of the cytokine microenvironment in the testis and risk the development of testicular cancer, all of which related to male infertility (Mehta et al., 2020; Tveito, 2020).

SARS-CoV-2 has been proved to exist in the uterus, ovaries, and placenta (Algarroba et al., 2020; Bian and The COVID-19 Pathology Team, 2020; Ferraiolo et al., 2020; Hosier et al., 2020). *ACE2* is expressed in female follicles, endometrium (Algarroba et al., 2020; Hosier et al., 2020), and throughout different developmental stages of preimplantation embryos. The co-expression level of *ACE2* and *TMPRSS2* is highest on Day 6 during the embryonic development in trophectoderm (TE) cells, indicating that TE cells may be relatively susceptible to SARS-CoV-2 during that time window. Thus, the potential risk of SARS-CoV-2 infection during embryo transfer process in clinical *in vitro* fertilization (IVF) should be properly evaluated.

*ACE2* expression in endometrium may allow SARS-CoV-2 to enter endometrial epithelial and stromal cells, impairing *in vivo* decidualization, embryo implantation, and placentation (Chadchan et al., 2020). *ACE2*-positive-expressing cells are also distributed at the maternal–fetal interface. The co-expression of *ACE2* and *TMPRSS2* mainly exists in the extravillous trophoblast cells (EVTs_24W) and syncytial trophoblast cells (STB_8W) of the decidua, making vertical transmission possible in the early and second trimester. Therefore, women with COVID-19 may have a higher risk of miscarriage (Chen et al., 2020b).

## Summary

SARS-CoV-2 infection has significant impacts on physical and mental health. In this review, potential effects of COVID-19 on human reproduction and the possibility of SARS-CoV-2 vertical transmission are discussed (Figure 1).

The incidence of infertility has been increasing recently, and miscarriage rate cannot be ignored in the natural pregnancy population. In the past few decades, although the success rate of infertility treatment has been greatly improved, the live birth rate of assisted reproductive technologies such as IVF still cannot exceed 50%. The above studies have confirmed that SARS-CoV-2 existed in human reproductive system of COVID-19 patients, and SARS-CoV-2 infection may affect sperm motility and T/LH and FSH/LH ratios. Thus, clarifying the impacts of SARS-CoV-2 infection on human reproduction will provide suggestions for people of childbearing age and construct a theoretical framework for IVF and embryo transfer process.

Pregnant women are susceptible to respiratory pathogens and may develop severe pneumonia. This makes them especially vulnerable for contracting SARS-CoV-2, even more so if they have chronic diseases or maternal complications. To enhance the protection for pregnant women, they should be informed about prenatal check-up items, check-up time intervals, and check-up content during pregnancy. It is not recommended for pregnant women to listen to fetal heart rate at home instead of having regular pregnancy check-up. During the epidemic, for women within 28 weeks of pregnancy, the time intervals for their prenatal check-ups can be appropriately extended if there is no noticeable discomfort in the first and second trimester. Women with over 28 weeks of pregnancy, however, should follow their doctor’s advice to go to the hospital for check-up. Pregnant women with chronic diseases or maternal complications should pay more attention to their health conditions. Pregnant women with severe or critical COVID-19 should consider terminating the pregnancy, and cesarean sections is recommended. Labor and delivery should be managed in a designated negative pressure room with experienced staff on personal protective equipment. Neonates should be isolated in a designated unit for at least 14 days after birth, and breastfeeding should be minimized to avoid close contact with the mother suspected or confirmed with COVID-19. Males and females are advised to engage in reproduction at least 3–6 months after recovering from COVID-19. More follow-up studies should be conducted to further evaluate the safety and health of pregnant women and newborns with COVID-19.

Currently, few studies pay attention to the long-term health status of neonates. More are tended to evaluate the status of newborns at birth using the Apgar score, which is mostly between 8–10 points (Schwartz, 2020; Wu et al., 2020; Yan et al., 2020; Yu et al., 2020). Although some cases suffered complications like premature birth, neonatal distress, and so on, none of the conditions has anything to do with COVID-19. A cohort study observed 116 COVID-19 pregnant women and conducted a follow-up on 82 neonates (Salvatore et al., 2020). None of the newborns showed symptoms of COVID-19. In addition, researchers from China also conducted prospective analysis of clinical characteristics and prognosis of 19 newborns from Wuhan (Liu et al., 2020b), monitoring the newborns’ vital signs, blood oxygen saturation, etc. The result indicated that the health status of the newborns was not affected. According to the present results, there is no evidence to support that pregnant women infected with SARS-CoV-2 would affect their neonates. To reach a concrete conclusion, further studies are needed.
